# Short-term ionic plasticity at GABAergic synapses

**DOI:** 10.3389/fnsyn.2012.00005

**Published:** 2012-10-16

**Authors:** Joseph V. Raimondo, Henry Markram, Colin J. Akerman

**Affiliations:** ^1^Akerman Lab, Department of Pharmacology, Oxford UniversityOxford, Oxfordshire, UK; ^2^Blue Brain Project, Brain Mind Institute, Ecole Polytechnique Fédérale de LausanneLausanne, Switzerland

**Keywords:** short-term ionic plasticity, GABA, chloride, GABA_A_ receptors, synaptic transmission, EGABA, reversal potential, pH

## Abstract

Fast synaptic inhibition in the brain is mediated by the pre-synaptic release of the neurotransmitter γ-Aminobutyric acid (GABA)and the post-synaptic activation of GABA-sensitive ionotropic receptors. As with excitatory synapses, it is being increasinly appreciated that a variety of plastic processes occur at inhibitory synapses, which operate over a range of timescales. Here we examine a form of activity-dependent plasticity that is somewhat unique to GABAergic transmission. This involves short-lasting changes to the ionic driving force for the post-synaptic receptors, a process referred to as short-term ionic plasticity. These changes are directly related to the history of activity at inhibitory synapses and are influenced by a variety of factors including the location of the synapse and the post-synaptic cell's ion regulation mechanisms. We explore the processes underlying this form of plasticity, when and where it can occur, and how it is likely to impact network activity.

## Introduction

Synaptic plasticity refers to structural and functional changes that occur at synapses in response to particular stimuli or patterns of activity. These processes can operate over a range of timescales, from short-term modification of synaptic transmission occurring over tens of milliseconds, to long-lasting changes that persist for hours and more. The changes that result are thought to contribute to phenomena as important and diverse as synaptic integration, the flow of information through neuronal circuits, learning and memory, neural circuit development and disease states such as epilepsy. In conjunction with the extensive research on plasticity at excitatory glutamatergic synapses, plastic processes at inhibitory synapses have received increasing attention over the past two decades. This reflects a growing appreciation of how fluctuations in the strength of inhibitory synapses also shape the input-output relationship of neurons and the computations of neuronal networks. This review focuses upon short-lasting changes in the strength of inhibitory synapses. Short-term plasticity has classically been linked to changes in vesicular release probability or mechanisms that alter post-synaptic conductance, such as receptor desensitization. In addition to these processes, inhibitory synapses exhibit a form of short-term plasticity that involves changes in the ionic driving force for post-synaptic ionotropic receptors. This process is the short-term variety of what has previously been referred to as ionic plasticity (Rivera et al., 2005; Blaesse et al., 2009) or ionic shift plasticity (Lamsa et al., 2010). Type A ionotropic γ-Aminobutyric acid receptors (GABA_A_Rs) are the primary mediators of fast synaptic inhibition in the brain and the reversal potential for these receptors (*E*_GABA_) is typically close to the neuronal resting membrane potential. This means that relatively small changes to *E*_GABA_ can vary the functional effect of GABA_A_R activation and consequently neuronal output. Here we explore how short-term, activity-dependent changes in the driving force for GABA_A_Rs occur and how they might affect ongoing physiological and pathological network activity.

## GABA_A_ receptor mediated synaptic transmission and plasticity

Two principle variables determine the effect of GABA_A_R mediated synaptic transmission on the post-synaptic membrane potential. The first is *E*_GABA_ and the second is the GABA_A_R conductance (*g*GABA). Open GABA_A_Rs are approximately four times more permeable to chloride (Cl^−^) than to bicarbonate (HCO^−^_3_) ions (Kaila and Voipio, 1987; Kaila et al., 1989). Therefore at rest, *E*_GABA_ (typically −75 mV) is much closer to the very negative Cl^−^ reversal (*E*_Cl^−^_; typically −85 mV) than the considerably more positive HCO^−^_3_ reversal (*E*_HCO^−^_3__; typically −20 mV) (Kaila et al., 1993; Lambert and Grover, 1995). When GABA binds to GABA_A_Rs, the bulk of anion flux through the channel is Cl^−^ flowing down its electrochemical gradient from outside to inside the cell. This causes the membrane potential hyperpolarization typical of classic GABA_A_R mediated inhibition. However, if *E*_Cl^−^_ is positive of the resting membrane potential, GABA_A_R activation will result in Cl^−^ efflux and depolarization. This can still have an inhibitory influence due to the shunting effect upon the membrane, and particularly if *E*_Cl^−^_ and *E*_GABA_ remain more negative than the action potential threshold (Kaila, 1994; Farrant and Kaila, 2007). If *E*_Cl^−^_ exceeds the action potential threshold, GABA_A_R mediated transmission is likely to increase the probability of action potential generation and will therefore exert an excitatory effect. In this manner the intracellular Cl^−^ concentration [Cl^−^]_i_, by setting *E*_Cl^−^_ and *E*_GABA_, determines the “mode” of GABA_A_R operation. The extent to which GABAergic inputs can move a neuron's membrane potential toward *E*_GABA_ is a function of *g*GABA. *g*GABA in turn is determined by a host of synaptic parameters including the amount of transmitter released, the number of GABA_A_Rs present, the GABA_A_R subunit composition, channel kinetics, phosphorylation state and presence of channel modulators. Whereas *E*_GABA_ sets the “mode,” *g*GABA can be thought of as determining the “strength” of the GABAergic synapse.

Changes to either *g*GABA or *E*_GABA_ are known to underlie long-term plasticity at GABAergic synapses (Gaiarsa et al., 2002; Wright et al., 2011). These sustained changes to GABAergic transmission have been demonstrated in numerous brain regions, species type and experimental preparations, and can be generated by periods of either pathological or physiological activity (Cohen et al., 2002; Woodin et al., 2003; Fiumelli et al., 2005; Pathak et al., 2007). In addition to long-term changes in GABAergic synaptic function, it is known that post-synaptic responses can also vary on short time scales, as a function of recent pre-synaptic activity (Davies et al., 1990; Fleidervish and Gutnick, 1995; Gupta et al., 2000). Repeated activation at some synapses can result in enhanced transmission (facilitation), while at other synapses repeated use results in a transient decrease in transmission (depression). In reality, multiple short-term plasticity mechanisms are likely to co-occur at synapses and the resulting behavior will be a combination of facilitation and depression that depends on the timing of synaptic activation (Tsodyks and Markram, 1997; Varela et al., 1997). Indeed, GABAergic synapses are known to display an array of short-term plasticity phenomena (Davies et al., 1990; Fleidervish and Gutnick, 1995; Jiang et al., 2000; Kirischuk et al., 2002; Mott et al., 1993), and in some cases have been related to the specific interneuron type that is the pre-synaptic source (Gupta et al., 2000; Pouille and Scanziani, 2004). Short-term plasticity phenomena such as these are generally understood to relate pre-dominantly to pre-synaptic processes. For instance, synaptic facilitation is typically attributed to residual elevations of pre-synaptic calcium (Ca^2+^), whilst synaptic depression is linked either to depletion of readily releasable synaptic vesicles (Zucker and Regehr, 2002) or the activation of pre-synaptic GABA_B_ receptors (Davies et al., 1990; Lambert and Wilson, 1994). However, post-synaptic mechanisms can also contribute to short-term synaptic plasticity at GABAergic synapses and these include desensitization of the post-synaptic receptors (McCarren and Alger, 1985; Overstreet et al., 2000) or changes in the ionic driving force for the post-synaptic receptors. This latter process—transient shifts in the ionic driving force of the post-synaptic GABA_A_Rs—will form the focus of the remainder of this review.

## The basic mechanism underlying short-term ionic plasticity at GABAergic synapses

Short-term changes to receptor reversal potentials via breakdown of ionic concentration gradients are not thought to occur at glutamatergic synapses. This is because the major ionotropic receptors for glutamate, AMPA, NMDA, and Kainate receptors, are equally permeable to Na^+^ and K^+^. The concentration gradients across the neuronal membrane for these two ions are diametrically opposed, resulting in a reversal potential for glutamate receptors of approximately 0 mV. During periods of intense glutamatergic synaptic activity, sodium influx and potassium efflux may reduce their respective local concentration gradients, but as both ion concentrations are perturbed to a similar extent, this will have a minimal effect on the combined reversal potential for glutamate receptors.

**Figure 1:**
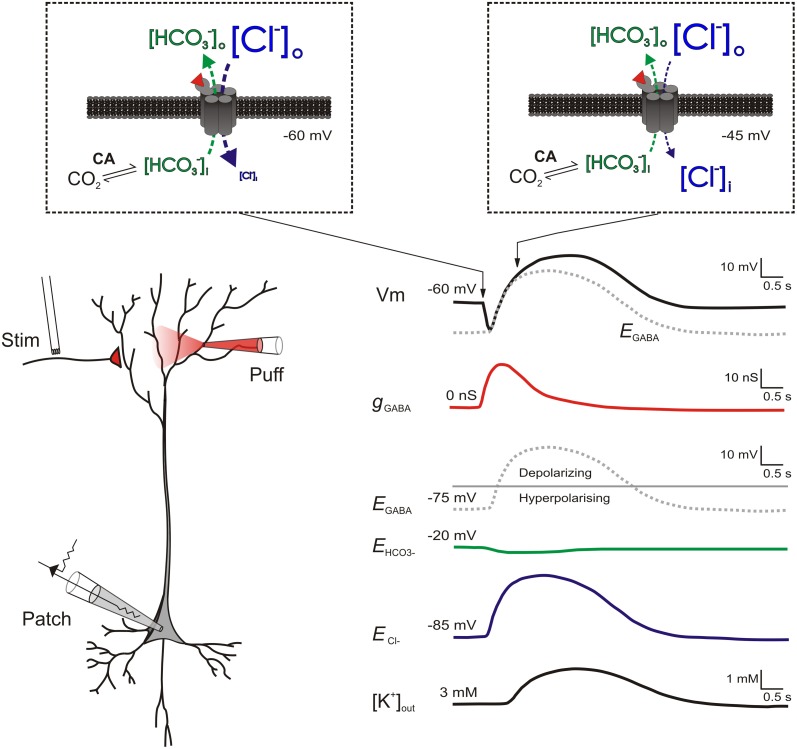
**Biphasic responses to intense GABA_A_R activation are caused by a rapid shift from hyperpolarizing to depolarizing *E*_GABA_.** (Left) a schematic of a patched pyramidal neuron receiving strong GABA_A_R input either via stimulation of GABAergic afferents or application of GABA. (Right) traces showing the putative changes in ionic and synaptic parameters as a result of the GABA_A_R activation. Separate traces show the cell's membrane potential (Vm, black); the GABA_A_R conductance (g_GABA_, red), the reversal potentials for the GABA_A_R (*E*_GABA_, gray dashed), HCO^−^_3_ (*E*_HCO_3__, green) and chloride (*E*_Cl^−^_, blue); plus the extracellular K+ concentration ([K^+^]_out_, black). Insets (within dashed boxes) show transmembrane ion fluxes and gradients at two points during the response to GABA_A_R activation. At the start of GABA_A_R activation (left inset) [Cl^−^] is typically much higher outside neurons (e.g., 135 mM) as opposed to inside neurons (e.g., 6 mM). In contrast, [HCO^−^_3_] is only moderately higher outside (23 mM) as compared to inside (12 mM). Therefore at a typical resting membrane potential of −60 mV, when GABA (red wedge) binds to ionotropic GABA_A_Rs, Cl^−^ flows into the cell (blue arrow) while HCO^−^_3_ flows out (green arrow). As GABA_A_Rs are approximately four times more permeable to Cl^−^ than to HCO^−^_3_ ions (Kaila and Voipio, 1987), the bulk of anion flux through the receptors is Cl^−^. This causes the membrane potential hyperpolarization typical of classic GABA_A_R mediated inhibition. With continued GABA_A_R activation (right inset), Cl^−^ influx ultimately exceeds Cl^−^ extrusion mechanisms and a reduction in the transmembrane Cl^−^ gradient occurs (Staley and Proctor, 1999). A corresponding depletion of intracellular HCO^−^_3_ is prevented by the activity of carbonic anhydrase, which uses CO_2_ as a substrate to rapidly regenerate HCO^−^_3_ (Rivera et al., 2005). As a result, *E*_Cl^−^_ (blue trace) and hence *E*_GABA_ shift toward the more positive *E*_HCO^−^_3__ (green trace) causing the membrane depolarization typical of the biphasic GABAergic response. Intracellular Cl^−^ accumulation also results in the delayed extrusion of K^+^ into the extracellular space via the Cl^−^/K^+^ cotransporter KCC2. This further contributes to the late-stage depolarization of the biphasic response (Kaila et al., 1997; Viitanen et al., 2010).

The situation within the GABAergic system is quite different. As described above, the major ionotropic receptor for GABA, the GABA_A_R, is permeable primarily to Cl^−^ and to a lesser extent HCO^−^_3_ (Kaila and Voipio, 1987; Kaila et al., 1989). Therefore at rest, *E*_GABA_ (typically −75 mV) is considerably closer to the very negative Cl^−^ reversal (*E*_Cl^−^_; typically −85 mV) than the more positive HCO^−^_3_ reversal (*E*_HCO^−^_3__; typically −20 mV) (Kaila et al., 1993; Lambert and Grover, 1995). During intense activation of GABA_A_Rs however, rapid Cl^−^ influx can exceed Cl^−^ extrusion mechanisms and a reduction in the transmembrane Cl^−^ gradient occurs (Kaila and Voipio, 1987; Kaila et al., 1989; Staley et al., 1995; Staley and Proctor, 1999). It is thought that a corresponding collapse of the HCO^−^_3_ gradient is prevented by the activity of intra- and extra-cellular carbonic anhydrases, which use CO_2_ as a substrate to rapidly regenerate intracellular HCO^−^_3_ (Kaila et al., 1990; Rivera et al., 2005). As a result, the intracellular Cl^−^ accumulation that occurs during repeated activation of GABA_A_Rs means that *E*_Cl^−^_ and hence *E*_GABA_ shift toward the more positive *E*_HCO^−^_3__ (Figure 1). Such a process is thought to contribute to short-term synaptic depression of GABAergic potentials (McCarren and Alger, 1985; Huguenard and Alger, 1986).

This process does not only reduce the size of inhibitory post-synaptic potentials (IPSPs), but strong GABA_A_R activation may cause IPSPs to change from being hyperpolarizing to depolarizing, as *E*_GABA_ shifts in a positive direction (Figure 1). Such biphasic responses have been widely documented (Alger and Nicoll, 1979; Andersen et al., 1980; Thompson and Gahwiler, 1989a,b). As described above, the most accepted explanation for this phenomenon is the differential collapse of the opposing concentration gradients of Cl^−^ and HCO^−^_3_ (Kaila et al., 1989; Staley et al., 1995; Staley and Proctor, 1999), although some contradictory observations remain unexplained (Perkins and Wong, 1997; Perkins, 1999). The magnitude of the late phase of the biphasic response is accentuated by extracellular potassium accumulation which further serves to depolarize the cell membrane (Kaila et al., 1997; Smirnov et al., 1999; Voipio and Kaila, 2000). This is thought to occur via the activity of the electroneutral K^+^-Cl^−^ co-transporter KCC2, which leads to the accelerated extrusion of both Cl^−^ and K^+^ in response to the GABA_A_R-mediated accumulation of intracellular Cl^−^ (Viitanen et al., 2010). It should be remembered that such shifts in *E*_GABA_ are expected to be temporary, which is why this phenomenon can be thought of as a short-term plastic change. Once GABA_A_R activity subsides, transporter proteins will return [Cl^−^]_i_ to resting levels on a timescale of seconds or minutes, depending upon the size of the *E*_GABA_ shift (Staley and Proctor, 1999; Raimondo et al., 2012a).

## Factors that influence ionic plasticity at GABAergic synapses

Any factor that affects the rate of Cl^−^ accumulation during GABA_A_R activation will influence how rapidly and by how much *E*_GABA_ shifts. For example, one would expect that the greater the Cl^−^ extruding capability of a neuron, the more resistant it would be to activity induced Cl^−^ accumulation. Several mechanisms have been identified to play a role in Cl^−^ efflux. These include Cl^−^ co-transporters such as KCC2 (Gamba, 2005), the Cl^−^/HCO^−^_3_ exchanger (Sterling and Casey, 1999) and voltage-sensitive Cl^−^ channels (Rinke et al., 2010). Despite these multiple potential Cl^−^ extruding pathways, electrophysiological experiments have shown that following a Cl^−^ load, it is possible to fit the recovery of [Cl^−^]_i_ with a single exponent (Staley and Proctor, 1999; Raimondo et al., 2012a). This suggests that a single transporter, described by a single exponential process, is likely to play a dominant role in the recovery from Cl^−^ accumulation. In most adult neurons, KCC2 has been identified as the major player in this process (Blaesse et al., 2009). As one would expect, reducing KCC2 activity within the context of a computational model (Doyon et al., 2011), or experimentally by genetic knockdown or pharmacological inhibition (Thompson and Gahwiler, 1989b; Jarolimek et al., 1999; Rivera et al., 2005; Zhu et al., 2005; Doyon et al., 2011), causes a depolarizing shift in resting *E*_GABA_. In addition, evidence suggests that reduced KCC2 activity hampers a neuron's ability to deal with an accumulation of intracellular Cl^−^ and therefore slows the time to recover normal synaptic inhibition (Jin et al., 2005; Doyon et al., 2011). What may be underappreciated is that KCC2 has a limited Cl^−^ affinity and transport capacity (Payne, 1997; Staley and Proctor, 1999; Song et al., 2002; Blaesse et al., 2009). This means that at a local level, and during the short time periods accompanying intense GABA_A_R activation, the volume of the compartment into which Cl^−^ flows and the rate of diffusion into other areas are also important parameters in governing the extent to which the Cl^−^ concentration increases intracellularly (Staley and Proctor, 1999; Doyon et al., 2011).

**Figure 2:**
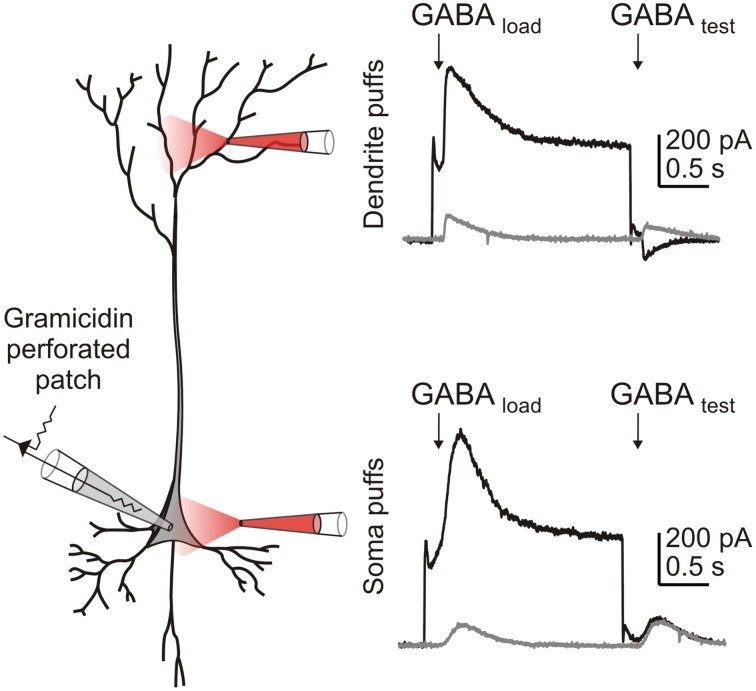
**Intense GABA_A_R activation results in Cl^−^ accumulation more readily in smaller volume compartments of the cell, than in larger volume compartments.** A CA3 hippocampal pyramidal cell within a P14 hippocampal slice culture was patched using the gramicidin perforated patch technique. HCO^−^_3_ was excluded from the external solution to ensure that GABA_A_R currents were purely attributable to Cl^−^. GABA_A_R activation was evoked by local application of 100 μM GABA to either the dendrites (top) or soma (bottom) of the neuron. In voltage clamp mode, Cl^−^ was loaded by stepping the membrane voltage to −37.5 mV during application of the first “loading” puff (“GABA load”), before returning to −60 mV for the second “test” puff (“GABA test”). When the puffer pipette was positioned over the dendrites, a Cl^−^ load affected the size and direction of the GABA_A_R current observed in response to the “test” puff. In contrast, this effect was not seen when a similar Cl^−^ load was generated at the soma.

Computational models (Qian and Sejnowski, 1990; Staley and Proctor, 1999; Doyon et al., 2011; Jedlicka et al., 2011) predict that for a given amount of synaptic GABA_A_R activation and its accompanying Cl^−^ influx, smaller post-synaptic volumes will result in relatively larger increases in [Cl^−^]_i_ and hence greater depolarizing shifts in *E*_GABA_. This explains the experimental finding that depolarizing responses to GABA_A_R activation are more easily elicited over dendritic as opposed to somatic compartments (Figure 2 and Staley and Proctor, 1999). In a theoretical paper, Qian and Sejnowski (1990) employed this reasoning to suggest that GABA_A_R-mediated inhibition is likely to be ineffective on dendritic spines. Due to their minute volume, even small amounts of Cl^−^ influx into a spine would be predicted to cause a local increase in [Cl^−^]_i_ that would rapidly depolarize *E*_GABA_. Consistent with this idea, it has since been confirmed that most GABAergic synapses are localized to dendritic shafts as opposed to spines (Freund and Buzsáki, 1996; Megías et al., 2001). In a similar vein, distal dendrites, apical tufts and the axon are also predicted to be prone to Cl^−^ accumulation effects (Doyon et al., 2011). In addition to their small volume, the narrow diameter of these processes means that longitudinal diffusion of Cl^−^ to other parts of the cell is severely restricted. This implies that multiple dendrite-targeting GABAergic inputs originating from a single pre-synaptic cell would have a larger inhibitory effect if the synapses are distributed throughout the dendritic tree, as opposed to being clustered along a single branch. Once again, such a morphological arrangement appears to be evident in different systems (Doyon et al., 2011; Jedlicka et al., 2011).

As well as the volume and rate of diffusion out of the intracellular compartment, another important factor that affects Cl^−^ accumulation during GABA_A_R activity is the presence, affinity and capacity of carbonic anhydrase. For example, Staley et al. (1995) have shown that blocking carbonic anhydrase, the enzyme that maintains intracellular levels of HCO^−^_3_, prevents a depolarizing response to strong GABA_A_R activation. In addition, Ruusuvuori et al. (2004) demonstrated that it is the developmental up-regulation of carbonic anhydrase VII by the end of the second postnatal week that permits the emergence of biphasic GABAergic potentials in response to high frequency stimulation in rat hippocampus. HCO^−^_3_ ions therefore provide a pivotal link between neuronal regulation of Cl^−^ and pH (Kaila et al., 1989, 1993; Doyon et al., 2011). For instance, although carbonic anhydrase activity is largely able to regenerate HCO^−^_3_ intracellularly in the face of GABA_A_R induced HCO^−^_3_ efflux, the additional generation of intracellular hydrogen that accompanies intense activity (Schwiening et al., 1993; Wang et al., 1994; Xiong et al., 2000; Raimondo et al., 2012b) will shift the equilibrium set point of the carbonic anhydrase catalyzed reaction of H_2_O and CO_2_, to HCO^−^_3_ and H^+^. Therefore activity induced acidification is predicted to cause a reduction in the available intracellular HCO^−^_3_ and hence a hyperpolarization of *E*_HCO^−^_3__. This would ultimately reduce the depolarization achievable by intense GABA_A_R activation in the face of progressive intracellular acidification. For instance, an acidic shift of 0.2 pH units would be predicted to generate a hyperpolarization of *E*_GABA_ on the order of 5 mV, although this remains to be verified experimentally (Kaila et al., 1990, 1993; Doyon et al., 2011). Finally, the membrane potential at which GABA_A_R activation occurs will determine the driving force for Cl^−^ influx. If GABAergic inputs occur synchronously with post-synaptic spiking or depolarizing glutamatergic EPSPs, this will increase the relative driving force for co-active GABA_A_Rs and greatly facilitate Cl^−^ accumulation and a positive shift in *E*_GABA_. Given the influence of the above mentioned parameters, it is perhaps not surprising that different cell types might differ in their susceptibility to Cl^−^ accumulation. For example, Lamsa and Taira (2003) found that high frequency trains of stimulation generate depolarizing shifts in the *E*_GABA_ of hippocampal interneurons. However, corresponding stimulation did not elicit similar changes in *E*_GABA_ within CA3 pyramidal neurons.

In addition to cell type differences, the maturational state of a neuron is also likely to impact GABAergic ionic plasticity as Cl^−^ homeostasis mechanisms are known to change during development. Compared to the Cl^−^ extruder KCC2, immature neurons tend to express relatively high levels of the Na^+^-K^+^-Cl^−^ co-transporter, NKCC1, which imports Cl^−^ into the cells. This means that [Cl^−^]_i_ is often significantly higher than in adult neurons (Ben-Ari, 2002). As a consequence, *E*_GABA_ is often depolarized relative to the resting membrane potential. Under these conditions it is predicted that repeated activation of GABA_A_Rs would actually lead to a cumulative *depletion* of [Cl^−^]_i_, which would hyperpolarize *E*_GABA_ and reduce excitability in young tissue (Marchetti et al., 2005). Consistent with this, activity-dependent depletion of [Cl^−^]_i_ has been demonstrated within the chick spinal cord and is implicated in the termination of spontaneous network events (Chub et al., 2006). In addition, repetitive intense GABA_A_R activation via exogenous application of GABA or muscimol in young rodent cortex and hippocampus has been shown to deplete [Cl^−^]_i_ and hyperpolarize *E*_GABA_ (Brumback and Staley, 2008; Kolbaev et al., 2011). At a more general level, this work confirms that a cell's Cl^−^ handling mechanisms have fundamental effects on the nature of ionic plasticity at GABAergic inputs.

Given the multiple factors that can influence short-term ionic plasticity at GABAergic synapses, it is important to consider how they might interact under different scenarios. For example, the axon initial segment (AIS) of pyramidal neurons is known to receive input almost exclusively from GABAergic axo-axonic cells. This subcellular compartment therefore offers an intriguing example of how the parameters described above might combine to generate [Cl^−^]_i_ accumulation or depletion effects. Firstly, the small volume of the AIS is predicted to amplify the effects of any Cl^−^ flux on transmembrane concentration gradients. Secondly, the AIS has been shown to express the Cl^−^ importer NKCC1, instead of the Cl^−^ extruder KCC2, which results in a relatively positive *E*_GABA_ at rest (Szabadics et al., 2006; Khirug et al., 2008; Woodruff et al., 2010). This compartment is therefore predicted to experience [Cl^−^]_i_ depletion in response to repeated GABAergic inputs at hyperpolarized membrane potentials. However, if GABAergic inputs were to arrive coincident with membrane potential depolarization, such as during action potential firing, rapid [Cl^−^]_i_ accumulation could still occur. As the AIS is the site of spike generation, dynamic, local changes to *E*_GABA_ could have a significant effect upon neuronal output. Future research is necessary to determine the existence and possible relevance of activity-dependent Cl^−^ fluxes within the AIS.

To date, activity-dependent shifts in *E*_GABA_, have been demonstrated in response to intense GABA_A_R activation, typically elicited either by exogenous application of GABA_A_R agonists or high frequency stimulation of GABAergic afferents. Evidence that activity driven, short-term changes in *E*_GABA_ could occur *in vivo* have come from studies of hyper-active network activity states, such as those generated in experimental models of epilepsy. It is thought that the intense activation of GABA_A_Rs in combination with the concurrent membrane depolarization that occurs during epileptiform activity can cause rapid Cl^−^ accumulation (Lamsa and Kaila, 1997; Isomura et al., 2003; Fujiwara-Tsukamoto et al., 2010; Ilie et al., 2012). Indeed, the resultant erosion of GABA_A_R mediated inhibition serves to initiate or exacerbate the hyperexcitability that is characteristic of epileptiform events (Lopantsev and Avoli, 1998; Lasztóczi et al., 2011; Ilie et al., 2012; Lillis et al., 2012). Beyond heightened network activity, it is currently an open question as to what sort of physiologically relevant activity patterns could lead to short-term changes to *E*_GABA_. Nonetheless, it is important to note that levels of [Cl^−^]_i_ accumulation appear to increase linearly with the intensity of GABA_A_R activation. Even relatively weak stimulation can produce small changes in [Cl^−^]_i_ (Isomura et al., 2003; Berglund et al., 2006).

## Functional effects of short-term ionic plasticity at GABAergic synapses

What functional effects do short-term alterations in *E*_GABA_ have in the context of evolving pathological and physiological network activity? As described above, large depolarizing shifts in *E*_GABA_ have been shown to play a role in exacerbating and sustaining epileptic seizures. In addition, it has been observed that high frequency stimulation, of the sort used to induce classic long-term potentiation (LTP) at glutamatergic synapses, is sufficient to induce GABA_A_R mediated depolarization (Thompson and Gahwiler, 1989a). This has led to the suggestion that one function of a short-term activity-dependent depolarizing shift in *E*_GABA_ is to modulate the Mg^2+^ block on NMDA receptors. This would suggest that the short-term GABAergic plasticity described above may play an important role in regulating NMDA-dependent mechanisms of synaptic plasticity (Staley et al., 1995).

By artificially setting the *E*_GABA_ of a neuron, one may investigate how physiologically plausible shifts in the ionic driving force for GABA_A_Rs may impact activity. This has been performed experimentally by dialyzing neurons during whole-cell recordings with internal solutions of set [Cl^−^]_i_, using dynamic clamp to simulate GABAergic inputs with different *E*_GABA_ values, or more recently using a light-activated Cl^−^ pump to load neurons with different amounts of Cl^−^ (Raimondo et al., 2012a). Studies performed in this manner have demonstrated that shifting *E*_GABA_ to moderately depolarizing values can result in enhanced spiking probability and reduced spike latencies in response to GABAergic inputs (Wang et al., 2001; Akerman and Cline, 2006; Saraga et al., 2008; Valeeva et al., 2010; Wright et al., 2011; Raimondo et al., 2012a). Computational models have also been used to study the impact that modest changes in *E*_GABA_ might have upon neural signaling. For instance, shifting the *E*_GABA_ in a model of a mature CA1 pyramidal neuron from −75 mV to −70 mV results in an increase in action potential firing frequency by approximately 40% (Saraga et al., 2008). Similarly, depolarizing shifts in inhibitory reversal potentials by as little as 10 mV can considerably shorten the duration of inhibitory inputs at the soma (Jean-Xavier et al., 2007). Small changes to *E*_GABA_ are more likely to be functionally significant when a fine balance exists between GABAergic inhibition and facilitation. For instance, in neocortical layer 5 neurons and dentate granule cells, *E*_GABA_ has been reported to lie at values more depolarized than the resting membrane potential, but below the action potential threshold (Kaila et al., 1993; Gulledge and Stuart, 2003; Chiang et al., 2012). This means that small changes in *E*_GABA_ could bidirectionally modulate neuronal firing rates and spike times (Morita et al., 2006; Chiang et al., 2012).

An intriguing possibility is that Cl^−^ accumulation might adjust the processing capacity of a neuron's dendritic tree, and in a manner that depends upon the amount of information flowing through a particular network (Viitanen, 2010). The mechanism would operate as follows. If *E*_GABA_ is more negative than the resting membrane potential, then hyperpolarizing GABAergic inputs would spread further in time and space than their underlying conductances (Gulledge and Stuart, 2003). During periods of enhanced activity however, synaptic inputs to dendrites would be predicted to cause modest Cl^−^ accumulation such that *E*_GABA_ moves toward the resting membrane potential, where GABAergic inputs will generate an exclusively shunting effect (Doyon et al., 2011; Jedlicka et al., 2011). Such a transition would serve to increase the spatial and temporal precision for integrating synaptic inputs, changing the fidelity of spike generation and effectively increasing the processing capacity of the dendritic compartment (London and Häusser, 2005; Viitanen, 2010). As such, short-term GABAergic plasticity involving shifts in *E*_GABA_ could allow a neuron to adjust the information processing capacity of its dendritic tree “on the fly,” to meet the varied computational demands of changing levels of neural activity. Given that interneurons are fundamental for the synchronization of neuronal networks (Whittington et al., 1995; Wang and Buzsáki, 1996), the activity dependent transition of inhibitory post-synaptic potentials from hyperpolarizing to shunting could also have important consequences for network oscillations. For instance, Vida et al. (2006), using a network model of interneurons, showed that shifting *E*_GABA_ from hyperpolarizing (−75 mV) to shunting (−55 mV) resulted in oscillations of markedly increased coherence and higher γ band frequency. Even larger shifts in *E*_GABA_ however, ultimately result in a loss of synchrony (Jedlicka and Backus, 2006). Considering the role of gamma oscillations in spike timing dependent plasticity and sensory processing (Paulsen and Sejnowski, 2000; Engel and Singer, 2001; Buzsáki and Draguhn, 2004), it is conceivable that the dynamic modulation of *E*_GABA_ may have consequences for information coding and memory processes (Buzsaki, 2006; Mu and Poo, 2006; Richards et al., 2010).

An important question that can be asked about any plasticity phenomenon is one of synapse specificity. Does a plasticity process affect individual synapses between pre- and post-synaptic neurons, multiple surrounding synapses, or every connection to the post-synaptic cell in question? Considering that GABAergic interneurons are a highly heterogeneous cell population involved in a diverse array of functions, from setting network oscillations to providing dynamic gain control (Freund and Buzsáki, 1996; Gabernet et al., 2005), *E*_GABA_ changes could be relevant over a range of spatial scales. At one extreme, one can consider whether Cl^−^ accumulations could be sufficiently compartmentalized so as to remain specific to a single GABAergic synapse, akin to Ca^2+^ compartmentalization within a single dendritic spine (Bloodgood and Sabatini, 2007; Yuste, 2011). Földy et al. (2010) provide suggestive evidence that [Cl^−^]_i_ may be regulated on an input specific level. They show selective expression of the voltage gated Cl^−^ channel, CLC2, at post-synaptic sites receiving input from parvalbumin-expressing basket cells (PVBCs), but not at post-synaptic sites receiving input from cholecystokinin-expressing basket cells (CCKBCs). The presence of this inward-rectifying Cl^−^ channel means that the synapses are able to extrude Cl^−^ at a faster rate in the face of intense GABA_A_R activation, whereas synapses without CLC2 show a greater propensity to exhibit shifts in *E*_GABA_. As Foldy et al. point out however, the somatodendritic distribution of PVBC and CCKBC synapses are somewhat different, which is likely to contribute to *E*_GABA_ differences (Földy et al., 2010). Nonetheless, this work demonstrates that ionic plasticity could be regulated according to synapse type, although the effects are not necessarily restricted to a single synapse. Indeed, modeling and experimental studies suggest that Cl^−^ diffuses rapidly between synapses, with accumulations often only remaining localized at the level of a dendritic branch (Doyon et al., 2011; Jedlicka et al., 2011). This would suggest that activity dependent Cl^−^ accumulations are likely to extend to surrounding synapses and therefore affect levels of inhibition in a spatially diffuse manner. Taken together, these studies highlight that future work is required to systematically explore the cell and synapse specific rules that influence short-term *E*_GABA_ changes.

## Conclusion

Like the glutamatergic system, GABAergic synapses may undergo a wide array of both short- and long-term plasticity phenomena that rely on alterations in pre-synaptic release and/or post-synaptic receptor conductance. However, the GABAergic system is somewhat unique in that its function can also be relatively easily modified via changes to the ionic driving force for the GABA_A_R and in a way that relates to the history of synaptic activity. It seems clear that *E*_GABA_ should not be assumed to be invariant across a neuron, it is a dynamic variable that evolves across both time and space as a function of varied patterns of neural activity. We anticipate that examining how this aspect of neuronal signaling contributes to network activity will provide fertile ground for future research.

### Conflict of interest statement

The authors declare that the research was conducted in the absence of any commercial or financial relationships that could be construed as a potential conflict of interest.
